# A Longitudinal Cohort Study of Body Mass Index and Childhood Exposure to Secondhand Tobacco Smoke and Air Pollution: The Southern California Children’s Health Study

**DOI:** 10.1289/ehp.1307031

**Published:** 2014-11-12

**Authors:** Rob McConnell, Ernest Shen, Frank D. Gilliland, Michael Jerrett, Jennifer Wolch, Chih-Chieh Chang, Frederick Lurmann, Kiros Berhane

**Affiliations:** 1 Department of Preventive Medicine, Keck School of Medicine, University of Southern California, Los Angeles, California, USA; 2 Department of Environmental Health, School of Public Health, University of California, Berkeley, Berkeley, California, USA; 3 Department of City and Regional Planning, College of Environmental Design, University of California, Berkeley, Berkeley, California, USA; 4 Sonoma Technology, Inc., Petaluma, California, USA

## Abstract

**Background:**

Childhood body mass index (BMI) and obesity prevalence have been associated with exposure to secondhand smoke (SHS), maternal smoking during pregnancy, and vehicular air pollution. There has been little previous study of joint BMI effects of air pollution and tobacco smoke exposure.

**Methods:**

Information on exposure to SHS and maternal smoking during pregnancy was collected on 3,318 participants at enrollment into the Southern California Children’s Health Study. At study entry at average age of 10 years, residential near-roadway pollution exposure (NRP) was estimated based on a line source dispersion model accounting for traffic volume, proximity, and meteorology. Lifetime exposure to tobacco smoke was assessed by parent questionnaire. Associations with subsequent BMI growth trajectory based on annual measurements and attained BMI at 18 years of age were assessed using a multilevel modeling strategy.

**Results:**

Maternal smoking during pregnancy was associated with estimated BMI growth over 8-year follow-up (0.72 kg/m^2^ higher; 95% CI: 0.14, 1.31) and attained BMI (1.14 kg/m^2^ higher; 95% CI: 0.66, 1.62). SHS exposure before enrollment was positively associated with BMI growth (0.81 kg/m^2^ higher; 95% CI: 0.36, 1.27) and attained BMI (1.23 kg/m^2^ higher; 95% CI: 0.86, 1.61). Growth and attained BMI increased with more smokers in the home. Compared with children without a history of SHS and NRP below the median, attained BMI was 0.80 kg/m^2^ higher (95% CI: 0.27, 1.32) with exposure to high NRP without SHS; 0.85 kg/m^2^ higher (95% CI: 0.43, 1.28) with low NRP and a history of SHS; and 2.15 kg/m^2^ higher (95% CI: 1.52, 2.77) with high NRP and a history of SHS (interaction *p*-value 0.007). These results suggest a synergistic effect.

**Conclusions:**

Our findings strengthen emerging evidence that exposure to tobacco smoke and NRP contribute to development of childhood obesity and suggest that combined exposures may have synergistic effects.

**Citation:**

McConnell R, Shen E, Gilliland FD, Jerrett M, Wolch J, Chang CC, Lurmann F, Berhane K. 2015. A longitudinal cohort study of body mass index and childhood exposure to secondhand tobacco smoke and air pollution: the Southern California Children’s Health Study. Environ Health Perspect 123:360–366; http://dx.doi.org/10.1289/ehp.1307031

## Introduction

Prevalence of obesity in U.S. children has been increasing for > 40 years [Centers for Disease Control and Prevention (CDC) 2011]. The current epidemic threatens the health of a generation of children because obesity and increased body mass index (BMI) in childhood are strong determinants of morbidity and premature mortality in adult life (Reilly and Kelly 2011; Tirosh et al. 2011). Beyond dietary factors and physical activity, a growing body of evidence indicates that environmental stressors may play a role in the etiology of childhood obesity (Blumberg et al. 2011).

Maternal smoking during pregnancy (hereafter referred to as *in utero* exposure) predicts subsequent childhood obesity (Ino 2010). *In utero* and secondhand tobacco smoke (SHS) exposure in children are correlated, but a few studies have found BMI, overweight, and obesity to be associated with SHS independent of *in utero* exposure (Griffiths et al. 2010; Raum et al. 2011). There is also emerging evidence that SHS exposure in adults is associated with obesity, as well as other metabolic abnormalities (Xie et al. 2010; Zhang et al. 2011). A recent study suggested that *in utero* exposure to another combustion source, ambient near-roadway pollution (NRP; estimated based on measured maternal exposure to polyaromatic hydrocarbons), is associated with increased BMI and obesity at 7 years of age (Rundle et al. 2012). However, to our knowledge there has been no previous prospective investigation in school children of joint obesogenic effects of tobacco smoke and air pollution exposure. Because these exposures are common, they have potentially significant clinical and public health impact.

The Southern California Children’s Health Study (CHS) collected information on *in utero* exposure to maternal smoking, exposure to SHS (before or at the time of enrollment), and exposure to ambient particles and NRP at the time of enrollment in a longitudinal study of BMI growth trajectory across adolescence and attained BMI at 18 years of age (Gauderman et al. 2007; Jerrett et al. 2010). We estimated the independent and joint effects of tobacco smoke and ambient exposure on BMI in the CHS.

## Methods

**Figure 1:**
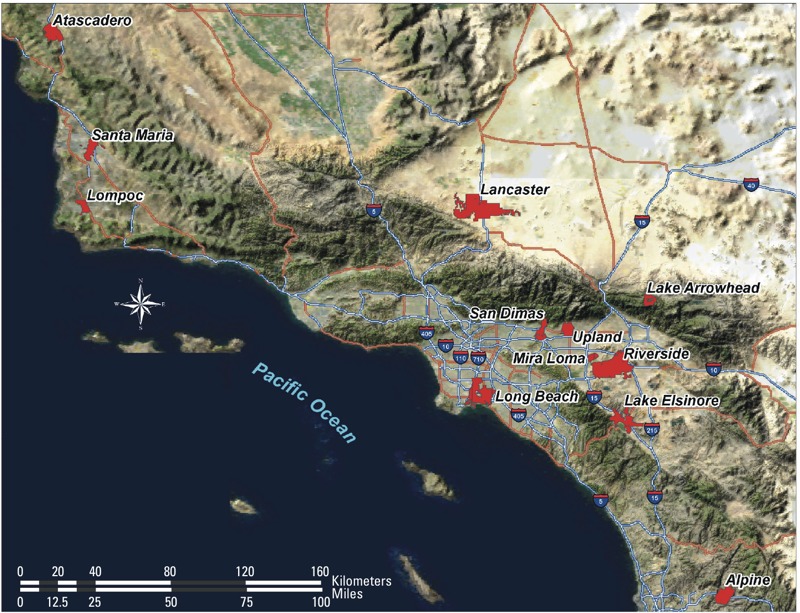
Southern California Children’s Health Study communities.

Two cohorts of children in fourth grade classrooms in 12 Southern California communities (Figure 1) were recruited at average age 10 years in 1993 and 1996 and followed through secondary school graduation 8 years later, as described previously (Gauderman et al. 2007; Jerrett et al. 2010). Of the 3,887 participants recruited, 569 (14.6%) had fewer than two BMI measurements, leaving a sample of 3,318 for analysis. Subsequent attrition was 5–10% per year. For analyses including NRP exposure, one community (*n* = 300 participants) was excluded, as described below.

Height (to the nearest centimeter) and weight (to the nearest pound) were measured at study entry and annually at the child’s school by a trained study technician, following a standardized procedure that included daily scale calibration (Jerrett et al. 2010). BMI (weight in kilograms divided by height in square meters) was calculated at each visit. Overweight and obesity were defined using the CDC criteria of > 85% and < 95%, and ≥ 95%, respectively, of the age- and sex-specific U.S. population distribution (CDC 2013). Participants in the analyses had on average (± SD) 6.4 ± 2.4 BMI measurements.

Parents completed a detailed baseline questionnaire that provided information about each child’s *in utero* and SHS exposure and relevant covariates, including physical activity, childhood medical history, and the child’s residential location history. Study procedures were approved by the University of Southern California Institutional Review Board, and parents provided written informed consent on behalf of themselves and their children at the time of enrollment of each child into the study.

Information on *in utero* exposure to maternal smoking, the child’s history of previous SHS exposure, and number of smokers in the home at study enrollment was provided in the baseline questionnaire. Children were classified as having *in utero* exposure if a parent indicated “yes” in response to the question “Did your child’s biologic mother smoke while she was pregnant with your child? (Include time when she was pregnant but did not yet know that she was.)” Any SHS exposure before study entry was classified based on a positive response to either of the following questions: “Does anyone living in this child’s home currently smoke cigarettes, cigars or pipes on a daily basis INSIDE THE HOME?” or “In the past, has anyone living in this child’s home ever smoked cigarettes on a daily basis INSIDE THE HOME while the child was living there?” For children exposed to SHS at home at study entry, the number of smokers was characterized by the question “How many people smoke inside this child’s home on a daily basis?” Responses were categorized as 0, 1, 2, or more smokers. Children who indicated that they had smoked > 6 cigarettes in the last week were classified as smokers.

*Near-roadway air pollution exposure*. Data on traffic proximity, volume, Southern California vehicular fleet emissions, and meteorology were used to characterize exposure at each child’s primary residential address at study entry. Participant residence and school addresses were standardized and their locations were geocoded to 13 m perpendicular to the side of the adjacent road, using the Tele Atlas database and software (http://www.na.teleatlas.com). Distance to the nearest major road was estimated using ESRI ArcGIS version 8.3 (http://www.esri.com). We included in the analysis only children with residential addresses for which the TeleAtlas geocoding software assigned its highest quality match code. These addresses were located on the correct side of the street, with their relative position between cross streets determined by linear interpolation of residence number between the nearest intersections. One of the original 12 CHS communities, Lake Arrowhead, was not included in analyses of NRP. Many participants in this community had only a post office box rather than a street address. Lake Arrowhead also has winding streets and long distances between intersections that made it difficult to interpolate accurately to a geocoded location.

Annual average daily traffic volumes on roadways were obtained from the California Department of Transportation Highway Performance Monitoring System (2005). Using previously described methods, we transferred the traffic volumes from the Department of Transportation roadway network to the TeleAtlas networks (Wu et al. 2005). Annual average exposure to local NRP at homes and schools was estimated from CALINE4 dispersion models that incorporate distance to roadways, vehicle counts, vehicle emission rates, wind speed and direction, and height of the mixing layer in each community (Benson 1992). Separate estimates were made for the contribution of traffic on all other roadways to concentrations of several pollutants, including carbon monoxide, nitrogen dioxide, total oxides of nitrogen (NO_x_), elemental and organic carbon, and particulate matter ≤ 10 μm (PM_10_) and ≤ 2.5 μm in aerodynamic diameter (PM_2.5_) These estimated pollutant exposures should be regarded as indicators of incremental increases due to primary emissions from local vehicular traffic on top of background ambient levels, based on annual average estimates. For our analysis, we characterized NRP exposure using modeled NO_x_ to represent the incremental contribution of local traffic to a more homogeneous community background concentration of NO_x_ that included both primary and secondary pollution resulting from long range transport and regional atmospheric photochemistry. This metric was highly correlated with other pollutants estimated by CALINE4 (*R* > 0.95). Therefore, modeled NO_x_ represented primary local NO_x_ from vehicular traffic, these other highly correlated pollutants in fresh traffic exhaust, and probably other pollutants for which we did not estimate exposures. The mean (± SD) NRP was 6.9 ± 7.5 (expressed as parts per billion of the near-roadway contribution to NO_x_), median 3.86 ppb. Associations with BMI were estimated for a 16.8-ppb increase in NRP, which represented the difference between the 90th and 10th percentiles of the overall distribution (17.8 and 1.0 ppb, respectively). Associations with quartiles of NRP exposure (< 1.85, 1.85–3.86, 3.86–9.08, and > 9.08 ppb) were also examined. In models of joint effects with SHS and *in utero* exposure, NRP was also treated as a dichotomous variable stratified at the study population median.

*Covariates*. We included child’s ethnicity and age, as reported by the parent on the baseline questionnaire at study entry, sex, community, and year of enrollment (1993 or 1996) in all models. In addition, we examined potential confounding by individual, household level, and neighborhood confounders of effects on BMI in this population, as described previously (Jerrett et al. 2010; Wolch et al. 2011). Information on child’s history of asthma and physical activity were based on information collected at study entry, including the number of team sports in which the child participated. Social characteristics of the child, family, and neighborhood included whether the child was born outside of the United States and had health insurance coverage at study entry, the educational attainment and responding parent preference for a Spanish questionnaire, neighborhood population density within 500 m of the child’s home, and residential census block unemployment among men and women and percentage living in poverty. Using information available from publicly and commercially available data sets, features of the built environment were obtained, including walkability based on street connectivity, acres of parks and recreation facilities, green space based on the Normalized Difference Vegetation Index (NDVI), and a restaurant or store selling food, all based on a 500-m Euclidian buffer around the child’s residence at study entry, using previously described methods (Jerrett et al. 2010; Wolch et al. 2011). Rates of violent crime in each study community were characterized, also as previously described (Jerrett et al. 2010; Shankardass et al. 2011; Wolch et al. 2011).

Information on annual average regional pollutant exposure to PM_10_ and PM_2.5_ mass, to nitrogen dioxide and to ozone from 1000 hours to 1800 hours was available from continuous measurements made at a central-site monitor in each community throughout the period of follow-up of this cohort. We have previously shown that there was relatively little temporal variation in the annual exposure to these pollutants in each community (Gauderman et al. 2004). Therefore, we used an 8-year annual average exposure to each regional pollutant for analyses.

*Statistical analysis*. We used a multilevel approach to estimate associations with BMI growth (i.e., the projected change in BMI) over an 8-year follow-up period while explicitly controlling for baseline BMI and for differences in the progression of BMI by age and sex. Sex-specific BMI trajectories were estimated using linear splines with knots at 12, 14, and 16 years of age to account for nonlinearity in BMI change, with random effects on all growth parameters to account for subject-to-subject heterogeneity in BMI trajectories. Models were parameterized to estimate associations of tobacco smoke and NRP with attained BMI at 18 years, in addition to associations with overall BMI growth over the follow-up period, and effects were estimated at various levels: between years, between individuals, and between communities.

The modeling approach is similar to what has been previously described (Berhane and Molitor 2008; Jerrett et al. 2010), but it includes a richer random-effects structure on all growth curve parameters for better control of subject-to-subject heterogeneity than we have previously used in analysis of this data set (Jerrett et al. 2010). We began by fitting a base model that included ethnicity, race, sex, year of enrollment, and community, with a missing indicator category used to account for missing values for potential confounders. Each potential confounder was examined to see whether it changed the coefficient of tobacco smoke or NRP exposure by at least 10% when added to the model. A missing indicator category was included for missing values for potential confounders. We also included a fixed or random effect for community, as appropriate. Because some regional pollutant concentrations were correlated with the community mean NRP exposure, NRP was deviated from its community mean to produce orthogonal exposures in models that also included regional pollution.

Because BMI trajectories during childhood are different for boys and girls, interaction terms were included in the final models, and Wald tests were used to assess whether effects of tobacco smoke and NRP exposure on BMI growth and attained level were different in boys and girls. Because they were not different, associations were reported for both sexes combined for parsimony. In addition, we modeled interactions between tobacco smoke and NRP exposures. In sensitivity analyses, we modeled the joint effects of NRP and SHS centered at each age of follow-up among 1,514 children at the same address for at least 4 years at study entry, to estimate associations with attained BMI in children with prolonged exposure to a consistent NRP environment before study enrollment.

Statistical significance was based on a probability of 0.05 for a two-sided hypothesis. Data were analyzed using SAS (version 9.2; SAS Institute Inc., Cary, NC) and R (version 3.0.1; R Foundation for Statistical Computing, Vienna, Austria) software. Data were compiled using ArcGIS 9.2, and some distance measurements were computed using Matlab version R2006a (Mathworks, Natick, MA).

## Results

The mean age of children at study entry in 1993 and 1996 in two waves of recruitment was 10.1 years (SD 0.59). Over the 8 years of follow-up from enrollment through year 8 (average age, 18 years), there was a 5.3-unit increase in mean BMI, from 18.3 ± 3.4 to 23.6 ± 5.1. The prevalence of obesity increased from 12% to 13.3% during this period.

Most children were non-Hispanic white or Hispanic (Table 1). Average BMI at baseline varied from 17.6 (± 2.9) kg/m^2^ among Asians to 19.1 (± 3.9) in Hispanics in this unadjusted analysis. A larger proportion of children had a history of SHS exposure (34.6%) at enrollment than of *in utero* exposure (16.7%). A history of exposure to SHS was associated with small increased BMI (18.5 ± 3.5 kg/m^2^) compared with children with no exposure (18.2 ± 3.4 kg/m^2^). Seven children reported personal smoking at study entry. Average BMI by quartile of NRP ranged from 18.1 ± 3.2 kg/m^2^ for the lowest quartile to 18.7 ± 3.6 kg/m^2^ for the second quartile, without evidence for a dose–response relationship. In addition, BMI was higher in children with asthma and in children who were foreign born, did not play a team sport, and who had a responding parent with less than a high school education or who completed a Spanish questionnaire (see Supplemental Material, Table S1).

**Table 1:** Selected participant characteristics and exposures at Children’s Health Study enrollment.

Characteristic	No. (%)^*a*^	BMI (mean ± SD)
Race/ethnicity
African American	155 (4.7)	18.7 ± 3.5
Asian	151 (4.5)	17.6 ± 2.9
Hispanic white	1,000 (30.1)	19.1 ± 3.9
Non-Hispanic white	1,825 (55.0)	18.0 ± 3.1
Other	187 (5.6)	18.3 ± 3.9
Sex
Male	1,647 (49.6)	18.4 ± 3.5
Female	1,671 (50.4)	18.3 ± 3.5
SHS at study entry
No one ever smoked in the house	2,070 (65.4)	18.2 ± 3.4
Anyone ever smoked in the house	1,094 (34.6)	18.5 ± 3.5
*In utero* exposure to maternal smoking
No	2,764 (83.2)	18.3 ± 3.5
Yes	554 (16.7)	18.6 ± 3.6
Personal smoking
No	3,311 (99.8)	18.4 ± 3.5
Yes	7 (0.21)	20.3 ± 4.7
NRP exposure^*b*^
Lowest quartile (< 1.85)	736	18.1 ± 3.2
Second quartile (1.85–3.86)	736	18.7 ± 3.6
Third quartile (> 3.86–9.08)	736	18.2 ± 3.5
Fourth quartile (> 9.08)	736	18.5 ± 3.8
^***a***^For the first observation of the participant (*n *= 3,318); denominator (*n *= 3,318) varies due to missing covariates values. ^***b***^In parts per billion NO_x_; 11 communities.		

Maternal smoking during pregnancy, a history of SHS exposure, and NRP exposure at the child’s home at study entry were each associated both with the estimated 8-year growth of BMI and with estimated attained BMI at 18 years (Table 2). Compared with children who had no history of SHS exposure at enrollment, those with SHS exposure had a greater estimated increase in BMI from baseline to age 18 years [0.81 kg/m^2^; 95% confidence interval (CI): 0.36, 1.27] and a higher attained BMI at age 18 (1.23 kg/m^2^ higher on average; 95% CI: 0.86, 1.61), consistent with a greater rate of increase during follow up combined with a higher average BMI at study entry. Number of smokers in the home at study entry was also associated with increased 8-year growth trajectory (0.48 kg/m^2^; 95% CI: 0.16, 1.12 for one smoker, 1.08 kg/m^2^; 95% CI: 0.19, 1.97 for two or more smokers) and with increased attained BMI at 18 years (0.95 kg/m^2^; 95% CI: 0.42, 1.47 for one smoker, 1.77 kg/m^2^; 95% CI: 1.04, 2.51 for two or more smokers). Associations of *in utero* exposure to maternal smoking (vs. no *in utero* exposure) with BMI growth (0.72 kg/m^2^ higher; 95% CI: 0.14, 1.31) and attained BMI at 18 years (1.14 kg/m^2^ higher; 95% CI: 0.66, 1.62) were similar in magnitude to associations of SHS with BMI. There was no evidence of an association between personal smoking at study entry with BMI growth (–0.33 kg/m^2^; 95% CI: –0.66, 1.61) and with attained level (–0.15 kg/m^2^; 95% CI: –0.40, 0.15), not tabulated. A 16.8-ppb increase in NRP (representing an increase from the 10th to the 90th percentile of the exposure distribution) also was associated with 8-year growth in BMI (1.13 kg/m^2^ higher; 95% CI: 0.61, 1.65) and attained BMI (1.27 kg/m^2^ higher; 95% CI: 0.75, 1.80; Table 2). When NRP exposure was categorized into quartiles, the association with BMI growth was 1.10 (95% CI: 0.46, 1.74), 1.26 (95% CI: 0.6, 1.91), and 2.31 (95% CI: 1.66, 2.96) kg/m^2^ for the 2nd, 3rd, and 4th quartile compared with the growth in children in the 1st quartile of exposure; attained BMI at 18 years was 1.63 (95% CI: 1.08, 2.18), 1.68 (95% CI: 1.08, 2.28), and 2.29 (95% CI: 1.57, 3.01) kg/m^2^ higher than in children in the 1st quartile of exposure (results not tabulated).

**Table 2:** Association of sources of tobacco smoke and NRP exposures^*a*^ at study enrollment with BMI growth over 8 years and with attained BMI at 18 years of age.

Exposure	BMI growth^*b*^ (95% CI)	Difference in attained BMI^*b*^ (95% CI)
SHS^*c*^	0.81 (0.36, 1.27)*	1.23 (0.86, 1.61)*
1 smoker in home	0.48 (0.16, 1.12)	0.95 (0.42, 1.47)
≥ 2 smokers in home	1.08 (0.19, 1.97)	1.77 (1.04, 2.51)
Maternal smoking during pregnancy^*c*^	0.72 (0.14, 1.31)	1.14 (0.66, 1.62)*
NRP^*c*^	1.13 (0.61, 1.65)*	1.27 (0.75, 1.80)*
^***a***^Exposure before or at enrollment at average age 10 years. ^***b***^BMI growth (kg/m^2^) over 8-year follow-up, and difference in attained BMI at age 18 years, compared with participants without tobacco smoke exposure, or scaled to NRP 10th–90th percentile range of 16.8 ppb of NO_x_, adjusted for ethnicity, sex, community, year of enrollment, and age; NRP exposure restricted to 11 communities. ^***c***^For SHS, *n* = 3,164; for maternal smoking during pregnancy, *n* = 3,318; for NRP, *n* = 2,944. *p < 0.001.		

In a model mutually adjusted for SHS and *in utero* exposure, the associations of each with BMI growth and attained BMI at age 18 were attenuated but remained significant, except for the adjusted effect estimate for *in utero* exposure on growth (0.35 kg/m^2^: 95% CI: –0.29, 0.99). (See Supplemental Material, Table S2.) However, SHS and *in utero* exposure to maternal smoking were correlated (phi coefficient 0.45).

There was no confounding (based on a change > 10% of the estimate in Table 2) of the associations of attained BMI at 18 years or of BMI growth with NRP, SHS, or *in utero* exposure to maternal smoking by team sports participation or history of asthma; by social characteristics of the child, family, or neighborhood, including whether the child was foreign born, parental education, language of questionnaire, residential population density, and residential census tract rates of unemployment; or by features of the built environment including neighborhood walkability, availability of sports and recreation facilities, and green cover. The effect of NRP was not confounded by community regional average PM_10_, PM_2.5_, ozone, or nitrogen dioxide levels (nor in these models were there statistically significant associations of any regional pollutant with BMI growth or attained level at 18 years of age; data not shown). However, there were modest changes in the estimates in Table 2 after adjusting for percent poverty in residential census tract, including increased effect of SHS (by 15%) on BMI growth to 0.93 kg/m^2^ (95% CI: 0.50, 1.36). Effect of NRP on growth was increased (by 12%) to 1.26 kg/m^2^ (95% CI: 0.62, 1.91). The effect of NRP on attained BMI level was reduced in models adjusted for availability of health insurance for the child (by 10%) to 1.14 kg/m^2^ (95% CI: 0.69, 1.59) and for a restaurant or food store within 500 m of the home (by 18%) to 1.04 kg/m^2^ (95% CI: 0.51, 1.56). In an analysis adjusting for a common set of these confounders across all models, associations of SHS exposure with BMI growth and attained BMI at 18 years of age were not substantially changed (see Supplemental Material, Table S3). There was modest attenuation of associations of NRP with BMI growth (from 1.13 kg/m^2^; 95% CI: 0.61, 1.65 to 0.99 kg/m^2^; 95% CI: 0.46, 1.51) and with attained BMI (from 1.27 kg/m^2^; 95% CI: 0.75, 1.80 to 1.10 kg/m^2^; 95% CI: 0.58, 1.61).

We also found no significant differences between boys and girls in the associations of tobacco smoke and NRP exposures with BMI growth or attained level, based on tests for interaction (data not shown).

We found evidence of synergism between tobacco smoke and NRP exposure on attained BMI at 18 years of age (Table 3). Compared with attained BMI among participants living in residences with low exposure to NRP with no history of SHS exposure, being in the upper half of the NRP exposure distribution (without a history of SHS) or having a history of SHS exposure (with low NRP) was associated with attained BMI estimates that were 0.80 kg/m^2^ higher (95% CI: 0.27, 1.32) and 0.85 kg/m^2^ higher (95% CI: 0.43, 1.28), respectively. Attained BMI among participants with both high NRP residences and a history of SHS exposure was 2.15 kg/m^2^ higher (95% CI: 1.52, 2.77) in contrast with an expected increase (based on the sum of the individual effect estimates) of 1.65 kg/m^2^ (interaction *p*-value = 0.007). There were no significant interactions of *in utero* exposure to maternal smoking with either NRP or SHS for BMI growth or attained level (data not shown).

**Table 3:** Synergistic effects of SHS and NRP exposures^*a*^ on attained BMI at age 18 years.

SHS	NRP exposure	Difference in attained BMI^*b*^(95% CI)
No	Low	Reference
No	High	0.80 (0.27, 1.32)*
Yes	Low	0.85 (0.43, 1.28)**
Yes	High	2.15 (1.52, 2.77)**
^***a***^Exposure before or at enrollment at average age 10 years (*n *= 2,806 with complete information on both exposures); NRP (11 communities) dichotomized at population median of NRP (3.86 ppb NO_x_). ^***b***^Difference in attained BMI (kg/m^2^) at age 18 years, compared with participants without SHS exposure and with low residential NRP exposure, adjusted for ethnicity, community, sex, year of enrollment, child health insurance, percent poverty in the census block, and no food outlet within 500-m road network residence buffer; analysis restricted to 11 communities. *p < 0.01. **p < 0.001.		

**Figure 2:**
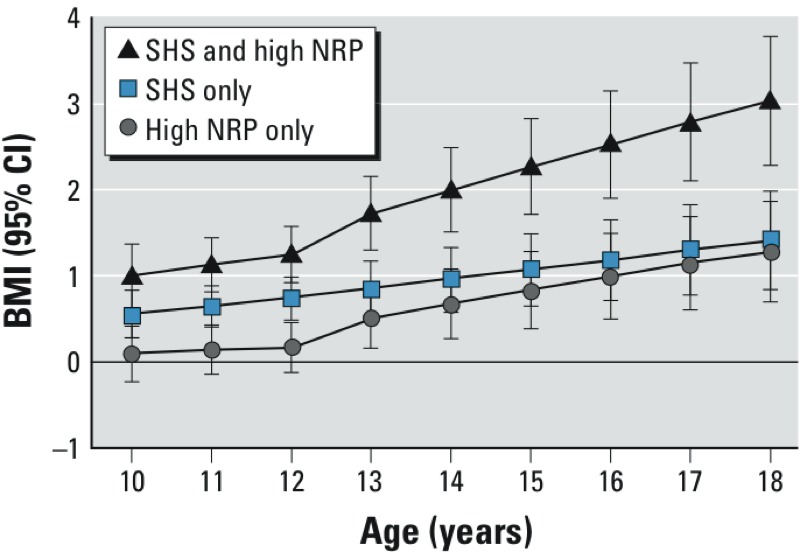
Estimated effects of SHS and NRP exposure on attained BMI at ages 10–18 years among long-term residents (*n *= 1,514 with ≥ 4 years at same residence at time of enrollment in 11 communities with NRP estimates). Differences in mean BMI (95% CIs) at each age were estimated for children with NRP above the median and no history of SHS (high NRP only), children with a history of SHS and NRP below the median (SHS only), and children with a history of SHS and high NRP, compared with a common reference group of children who had low NRP and no history of SHS at enrollment.

A sensitivity analysis was restricted to the 1,514 participants living at the same address for at least 4 years before study entry in order to determine whether effects would be larger in children with consistent longer-term NRP exposures. For this analysis the association of each exposure category with mean attained BMI was compared with BMI among referent children with neither SHS nor high residential NRP exposure (represented by zero on the *x*-axis in Figure 2). To illustrate the pattern of estimated effect on attained BMI the model-based estimate was re-centered at each age of follow-up. The association of attained BMI at 18 years with NRP with early-life SHS exposure was larger than that observed in the entire population (in Table 3), and the joint effect estimate accounted for almost an additional 3 kg/m^2^, compared with children who had neither exposure.

There was also evidence of an interaction between SHS and NRP based on NO_x_ modeled as a continuous variable. Specifically, among children with no history of SHS, a 16.8-ppb increase in NO_x_ was associated with a 0.34-kg/m^2^ increase in attained BMI at age 18 years (95% CI: –0.26, 0.94), compared with a 1.22-kg/m^2^ increase (95% CI: 0.65, 1.77) among children with a history of SHS (interaction *p*-value < 0.001).

## Discussion

Previous studies have reported associations between SHS and BMI in younger children (Griffiths et al. 2010; Raum et al. 2011). In our study population, both history of SHS exposure at study entry and of *in utero* exposure were associated with greater subsequent BMI growth over an 8-year period spanning adolescence through young adulthood. Furthermore, associations of SHS exposure with BMI attained by the time these children reached adult age were stronger among children exposed to higher levels of NRP. Children with a history of SHS exposure who lived in a home in the upper half of the NRP distribution had BMI at 18 years of age > 2 kg/m^2^ larger than BMI in young adults with neither SHS nor high NRP exposures. The prospective design and corresponding temporal sequence of exposure and substantial increases in BMI, which were not explained by a variety of potential confounders, the consistency of association and the synergism between combustion products from two different sources, and the emerging epidemiological and toxicological evidence all suggest that these combustion products contribute to the development of obesity.

Previous work has identified the systemic inflammatory effect of SHS and particulate air pollution as a potential common mechanism explaining the epidemiologic associations with cardiopulmonary disease [Pope and Dockery 2006; U.S. Department of Health and Human Services (DHHS) 2010]. Emerging evidence from experiments on mice suggests that PM_2.5_-induced adipose tissue inflammation and redistribution of adipose tissue to viscera may play a key role in the development of insulin resistance, diabetes, and systemic inflammatory effects (Sun et al. 2009; Zou 2010). However, in these studies metabolic and inflammatory effects of PM_2.5_ were not associated with weight gain. In contrast, a recent experimental study reported that early-life exposure of mice to diesel exhaust particulate, a model NRP, resulted in markedly larger subsequent weight gain than in animals exposed to filtered air (Bolton et al. 2012). Our results showing associations of BMI with NRP but not with regional PM are consistent with these experimental results. Further investigation is needed to understand the mechanisms underlying the NRP mixture effects.

Other biologically plausible explanations for the obesogenic effects of tobacco smoke and NRP include inhibition of catecholamine-induced lipolysis by polycyclic aromatic hydrocarbons (PAHs), resulting in weight gain in experimental animals (in the absence of increased caloric intake) (Irigaray et al. 2006). PAHs are part of the pollutant mixture in both NRP and SHS (DHHS 2010). Nicotine exposure from maternal smoking during pregnancy is a plausible cause of subsequent obesity, based on toxicological studies (Thayer et al. 2012). PM exposure has been reported to cause oxidative stress–mediated damage to mitochondria and down-regulation of expression of key brown adipose tissue genes involved in thermogenesis and energy expenditure (Xu et al. 2011). Direct inflammatory effects on feeding centers in the hypothalamus, or indirect effects through up-regulation of inflammatory pathways in adipose tissue, may also result in increased dietary intake (Bolton et al. 2012).

*In utero* exposure to tobacco smoke has been consistently reported to have obesogenic effects (Thayer et al. 2012). Our results support a few other studies showing increased BMI and obesity in SHS-exposed children, even after adjustment for *in utero* exposure (Griffiths et al. 2010; Raum et al. 2011). A few other epidemiological studies also have reported associations of markers of NRP exposure with obesity. Early-life exposure to PAHs in children in New York City was associated with the subsequent development of obesity by 7 years of age (Rundle et al. 2012).

We have previously reported associations between traffic density and BMI in this cohort, which we attributed to reduced opportunities for physical activity in an environment with heavy traffic corridors, although reported physical activity did not explain the associations (Jerrett et al. 2010). Exposure assignment to residence in the current analysis accounting for meteorology and vehicular emissions is likely to have been a more accurate estimate of NRP exposure than traffic density alone. Associations of modeled NRP exposure with increased BMI have also been observed in another Children’s Health Study cohort (Jerrett et al. 2014).

Maternal smoking and childhood SHS exposures may have been misclassified, although there is evidence from at least some populations that such exposures may be accurately reported by mothers (Gehring et al. 2006; Matt et al. 2000; Pickett et al. 2009). Exposure would have to have been both misclassified and also overreported by parents of children who would go on to have steeper BMI trajectory to explain the observed results. Given the strength of the association and the interaction with NRP, this seems an unlikely explanation for the observed results.

We examined the potential for selection or confounding bias to explain these results. There were 569 participants from the cohort who were not available for at least two measurements of height and weight. Attrition is a potential source of bias in a cohort study if loss to follow-up is related both to exposure and outcome, but BMI outcome at study entry did not differ between those lost to follow-up (18.5 ± 3.6 kg/m^2^) and those with two or more BMI measurements contributing to the analysis (18.3 ± 3.4 kg/m^2^). Mean exposure to NRP (adjusted for community) differed little between participants and those lost to follow-up (by only 0.013 ppb NO_x_). SHS exposure was more common among those lost to follow-up (45%) than in participants contributing to the analysis (35%), as was *in utero* exposure to maternal smoking (23% compared with 17%). Some other sociodemographic characteristics (foreign-born parents, health insurance for the child, race/ethnicity, and parental education) also differed among participants and those lost to follow-up (see Supplemental Material, Table S4). However, we adjusted for these sociodemographic characteristics, for health and physical activity information about the child, and for other features of the urban environment that might affect patterns of activity or food intake, and these did not substantially influence the pattern of associations of SHS and NRP with BMI. For caloric intake to explain our results, it would not only have had to be correlated both with SHS and NRP, but also to explain the synergistic pattern of association of these exposures with BMI, and this seems an unlikely confounding scenario. In addition, we observed significant associations of SHS and NRP with BMI in a subgroup of children who were followed through at least one of the last 2 years of the study (years 7 and 8 of follow-up; *n* = 2,120), and effect estimates were generally similar to those in the participants as a whole (results not shown). Although selection bias and confounding by other factors cannot be excluded as an explanation for our results, these analyses provide little evidence that this was the case.

The magnitude of the associations of BMI with SHS and NRP was large, especially in combination. The 2 kg/m^2^ associated with the combination of SHS with high-NRP exposures at 18 years of age (compared with low NRP and no SHS) were almost half the standard deviation of 5.1. A 2-kg/m^2^ relative increase in attained BMI is equivalent to a 6-kg (6.6%) increase in body weight in an adult male who is 1.78 m tall and weighs 95 kg (BMI = 30). For comparison, multifaceted, albeit short-term, interventions in adolescents are estimated to result in weight reductions of < 0.1 kg/m^2^ (Waters et al. 2011).

Further research is warranted to determine whether our findings can be replicated in other populations and to assess both the potential contribution of combustion sources to the epidemic of obesity and the potential impact of interventions to reduce exposure. *In utero* and SHS exposure and regional ambient PM exposure have been decreasing in California over the past several decades during which the obesity epidemic has developed (Al-Delaimy et al. 2010), so these exposures by themselves are unlikely to have contributed to the epidemic. However, these trends in regional air pollutants have limited relevance to the potential role of NRP, which is a different pollutant mixture. Vehicle miles traveled, exposure to some components of the NRP mixture, and near-roadway residential development have increased during this period (Geller et al. 2005) (U. S. Department of Transportation, Federal Highway Administration, Office of Highway Policy Information 2010). Thus, the potential for NRP to be among several factors contributing to the epidemic of obesity merits further investigation.

## Supplemental Material

(441 KB) PDF

## References

[r1] Al-Delaimy WK, White MM, Mills AL, Pierce JP, Emory K, Boman M, et al. (2010). Two Decades of the California Tobacco Control Program: California Tobacco Survey, 1990–2008. La Jolla, CA:University of California, San Diego.. http://www.cdph.ca.gov/programs/tobacco/Documents/CDPH_CTS2008%20summary%20report_final.pdf.

[r2] Benson P (1992). A review of the development and application of the Caline3 and Caline4 models.. Atmos Environ Part B Urban Atmosphere.

[r3] Berhane K, Molitor NT (2008). A Bayesian approach to functional based multi-level modeling of longitudinal data: with applications to environmental epidemiology.. Biostatistics.

[r4] Blumberg B, Iguchi T, Odermatt A (2011). Endocrine disrupting chemicals.. J Steroid Biochem Mol Biol.

[r5] Bolton JL, Smith SH, Huff NC, Gilmour MI, Foster WM, Auten RL (2012). Prenatal air pollution exposure induces neuroinflammation and predisposes offspring to weight gain in adulthood in a sex-specific manner.. FASEB J.

[r6] California Department of Transportation Highway Performance Monitoring System. (2005). California Motor Vehicle Stock, Travel and Fuel Forecast. Sacramento, CA:California Department of Transportation.. http://ntl.bts.gov/lib/24000/24000/24028/mvstaff05.pdf.

[r7] CDC (Centers for Disease Control and Prevention). (2011). CDC grand rounds: childhood obesity in the United States.. MMWR Morb Mortal Wkly Rep.

[r8] CDC (Centers for Disease Control and Prevention). (2013). Growth Chart Training Course.. http://www.cdc.gov/nccdphp/dnpao/growthcharts/index.htm.

[r9] DHHS (U.S. Department of Health and Human Services). (2010). How Tobacco Smoke Causes Disease: The Biology and Behavioral Basis for Smoking-Attributable Disease: A Report of the Surgeon General.

[r10] Gauderman WJ, Avol E, Gilliland F, Vora H, Thomas D, Berhane K (2004). The effect of air pollution on lung development from 10 to 18 years of age.. N Engl J Med.

[r11] Gauderman WJ, Vora H, McConnell R, Berhane K, Gilliland F, Thomas D (2007). Effect of exposure to traffic on lung development from 10 to 18 years of age: a cohort study.. Lancet.

[r12] Gehring U, Leaderer BP, Heinrich J, Oldenwening M, Giovannangelo ME, Nordling E (2006). Comparison of parental reports of smoking and residential air nicotine concentrations in children.. Occup Environ Med.

[r13] Geller MD, Sardar SB, Phuleria H, Fine PM, Sioutas C (2005). Measurements of particle number and mass concentrations and size distributions in a tunnel environment.. Environ Sci Technol.

[r14] Griffiths LJ, Hawkins SS, Cole TJ, Dezateux C, Millennium Cohort Study Child Health Group. (2010). Risk factors for rapid weight gain in preschool children: findings from a UK-wide prospective study.. Int J Obes (Lond).

[r15] Ino T (2010). Maternal smoking during pregnancy and offspring obesity: meta-analysis.. Pediatr Int.

[r16] Irigaray P, Ogier V, Jacquenet S, Notet V, Sibille P, Méjean L (2006). Benzo[*a*]pyrene impairs beta-adrenergic stimulation of adipose tissue lipolysis and causes weight gain in mice. A novel molecular mechanism of toxicity for a common food pollutant.. FEBS J.

[r17] Jerrett M, McConnell R, Chang CC, Wolch J, Reynolds K, Lurmann F (2010). Automobile traffic around the home and attained body mass index: a longitudinal cohort study of children aged 10–18 years.. Prev Med.

[r18] Jerrett M McConnell R Wolch J Chang R Lam C Dunton G 2014 Traffic-related air pollution and obesity formation in children: a longitudinal, multilevel analysis. Environ Health 13 1 49; 10.1186/1476-069X-13-49 24913018 PMC4106205

[r19] Matt GE, Hovell MF, Zakarian JM, Bernert JT, Pirkle JL, Hammond SK (2000). Measuring secondhand smoke exposure in babies: the reliability and validity of mother reports in a sample of low-income families.. Health Psychology.

[r20] Pickett KE, Kasza K, Biesecker G, Wright RJ, Wakschlag LS (2009). Women who remember, women who do not: a methodological study of maternal recall of smoking in pregnancy.. Nicotine Tob Res.

[r21] Pope CA, Dockery DW (2006). Health effects of fine particulate air pollution: lines that connect.. J Air Waste Manag Assoc.

[r22] Raum E, Küpper-Nybelen J, Lamerz A, Hebebrand J, Herpertz-Dahlmann B, Brenner H (2011). Tobacco smoke exposure before, during, and after pregnancy and risk of overweight at age 6.. Obesity (Silver Spring).

[r23] Reilly JJ, Kelly J (2011). Long-term impact of overweight and obesity in childhood and adolescence on morbidity and premature mortality in adulthood: systematic review.. Int J Obes (Lond).

[r24] Rundle A, Hoepner L, Hassoun A, Oberfield S, Freyer G, Holmes D (2012). Association of childhood obesity with maternal exposure to ambient air polycyclic aromatic hydrocarbons during pregnancy.. Am J Epidemiol.

[r25] Shankardass K, Jerrett M, Milam J, Richardson J, Berhane K, McConnell R (2011). Social environment and asthma: associations with crime and No Child Left Behind programmes.. J Epidemiol Community Health.

[r26] Sun Q, Yue P, Deiuliis JA, Lumeng CN, Kampfrath T, Mikolaj MB (2009). Ambient air pollution exaggerates adipose inflammation and insulin resistance in a mouse model of diet-induced obesity.. Circulation.

[r27] Thayer KA Heindel JJ Bucher JR Gallo MA 2012 Role of environmental chemicals in diabetes and obesity: a National Toxicology Program workshop review. Environ Health Perspect 120 6 779 789; 10.1289/ehp.1104597 22296744 PMC3385443

[r28] Tirosh A, Shai I, Afek A, Dubnov-Raz G, Ayalon N, Gordon B (2011). Adolescent BMI trajectory and risk of diabetes versus coronary disease.. N Eng J Med.

[r29] U.S. Department of Transportation, Federal Highway Administration, Office of Highway Policy Information. (2010). Highway Statistics 2010.

[r30] Waters E de Silva-Sanigorski A Hall BJ Brown T Campbell KJ Gao Y 2011 Interventions for preventing obesity in children. Cochrane Database Syst Rev 12 CD001871; 10.1002/14651858.CD001871.pub3 22161367

[r31] Wolch J, Jerrett M, Reynolds K, McConnell R, Chang R, Dahmann N (2011). Childhood obesity and proximity to urban parks and recreational resources: a longitudinal cohort study.. Health Place.

[r32] Wu J, Funk TH, Lurmann FW, Winer AM (2005). Improving spatial accuracy of roadway networks and geocoded addresses.. Transactions in GIS.

[r33] Xie B, Palmer PH, Pang Z, Sun P, Duan H, Johnson CA (2010). Environmental tobacco use and indicators of metabolic syndrome in Chinese adults.. Nicotine Tob Res.

[r34] Xu Z Xu X Zhong M Hotchkiss IP Lewandowski RP Wagner JG 2011 Ambient particulate air pollution induces oxidative stress and alterations of mitochondria and gene expression in brown and white adipose tissues. Part Fibre Toxicol 8 20; 10.1186/1743-8977-8-20 21745393 PMC3152885

[r35] Zhang L, Curhan GC, Hu FB, Rimm EB, Forman JP (2011). Association between passive and active smoking and incident type 2 diabetes in women.. Diabetes Care.

[r36] Zou MH (2010). Is NAD(P)H oxidase a missing link for air pollution-enhanced obesity?. Arterioscler Thromb Vasc Biol.

