# Erratum: A Longitudinal Cohort Study of Body Mass Index and Childhood Exposure to Secondhand Tobacco Smoke and Air Pollution: The Southern California Children’s Health Study

**DOI:** 10.1289/ehp.123-A81

**Published:** 2015-04-01

McConnell R, Shen E, Gilliland FD, Jerrett M, Wolch J, Chang CC, et al. 2015. Environ Health Perspect 123(4):360–366; http://dx.doi.org/10.1289/ehp.1307031

In Table S4 of the Supplemental Material, numbers of observations (N) and corresponding percentages (%) were mislabeled as mean values (kg/m^2^) ± SD. The table has been corrected, and the online PDF reflects these changes.

The authors regret the errors.

