# Supplementary material for: A Longitudinal Cohort Study of Body Mass Index and Childhood Exposure to Secondhand Tobacco Smoke and Air Pollution: The Southern California Children’s Health Study
Source: Environ Health Perspect. 2014 Nov 12;123(4):360–6. doi: 10.1289/ehp.1307031 (PMC4384197; doi:10.1289/ehp.1307031)
Supplement: (441 KB) PDF [file ehp.1307031.s001.508.pdf]

Environmental Health Perspectives doi:10.1289/ehp.1307031

Supplemental Material

A Longitudinal Cohort Study of Body Mass Index and Childhood Exposure to Secondhand Tobacco Smoke and Air Pollution: The Southern California Children's Health Study

Rob McConnell, Ernest Shen, Frank D. Gilliland, Michael Jerrett, Jennifer Wolch, Chih-Chieh Chang, Frederick Lurmann, and Kiros Berhane

Table S1. Selected personal, household and neighborhood characteristics of participant residences at study entry.

Characteristic	No. (%) or mean ± SD[a]	BMI mean ± SD (kg/m^2)
**Individual and household characteristics**		
Ever asthma		
No	2775 (85.8)	18.3 ± 3.4
Yes	459 (14.2)	18.8 ± 3.9
Play team sports		
No	1475 (46.0)	18.5 ± 3.7
Yes	1731 (54.0)	18.2 ± 3.3
Health insurance for child		
No	462 (15.0)	18.7 ± 3.7
Yes	2629 (85.0)	18.3 ± 3.4
Foreign born		
No	3059 (93.4)	18.3 ± 3.5
Yes	216 (6.6)	18.5 ± 3.4
Responding parental education		
High school or less	1055 (33.2)	18.6 ± 3.6
Some college	1417 (44.6)	18.2 ± 3.5
College	705 (22.2)	18.1 ± 3.1
Language of questionnaire		
Spanish	175 (5.5)	19.2 ± 3.9
English	2998 (94.5)	18.3 ± 3.4
**Local environment**		
Having a restaurant or food store within 500 m road network buffer		
No	2333 (73.8)	18.2 ± 3.3
Yes	826 (26.2)	18.9 ± 3.9
Population density (in 500m buffer)	1256 ± 1121	
Proportion unemployed in census block of home	0.05 ± 0.04	
Proportion in poverty in census block of home	0.08 ± 0.09	
Parks and recreation (acres in 500 m buffer)	5.6 ± 13.9	
Normalized Difference Vegetation Index (in 500 m buffer)	0.06 ± 0.08	

[a]For the first observation of the participant (N=3318); denominator varies due to missing covariates values.

Table S2. Associations of mutually adjusted secondhand (SHS) and in utero exposures[a] to tobacco smoke with BMI growth over 8 years and with attained BMI at age 18.

Exposure	BMI growth[b] (95% CI)	Difference in attained BMI[b] (95% CI)
SHS	0.81 (0.36, 1.27)[d]	1.23 (0.86, 1.60)[d]
SHS adjusted for *in utero* exposure	0.69 (0.19, 1.20)[c]	1,04 (0.62, 1.45)[d]
In utero exposure	0.74 (0.16, 1.32)	1.15 (0.67, 1.63)[d]
In utero exposure adjusted for SHS	0.35 (-0.29, 0.99)	0.58 (0.05, 1.11)

[a]Exposure prior to or at enrollment at average age 10; (N=3164 with information on both exposures). [b]BMI growth (in kg/m^2) over 8-year follow-up, and difference in attained BMI at age 18, compared with participants without tobacco smoke exposure, adjusted for ethnicity, gender, community, year of enrollment, and age. [c]$P<0.01$. [d]$P<0.001$.

Table S3. Fully adjusted associations of sources of tobacco smoke and near-roadway pollution (NRP) exposures[a] at study enrollment with BMI growth over 8 years and with attained BMI at age 18.

Exposure	BMI growth[b] (95% CI) From Table 2	BMI growth[c] (95% CI) Fully Adjusted	Difference in attained BMI[b] (95% CI) From Table 2	Difference in attained BMI[c] (95% CI) Fully Adjusted
SHS[d]	0.81 (0.36, 1.27)[d]	0.80 (0.35, 1.26)[e]	1.23 (0.86, 1.61)[e]	1.18 (0.81, 1.55)[e]
Maternal smoking during pregnancy[d]	0.72 (0.14, 1.31)[d]	0.72 (-0.14, 1.29)[e]	1.14 (0.66, 1.61)[e]	1.10 (0.62, 1.57)[e]
NRP[d]	1.13 (0.61, 1.65)[d]	0.99 (0.46, 1.51)[e]	1.27 (0.75, 1.80)[e]	1.10 (0.58, 1.61)[e]

[a]Exposure prior to or at enrollment at average age 10. [b]BMI growth (in kg/m^2) over 8-year follow-up, and difference in attained BMI at age 18, compared with participants without tobacco smoke exposure, or scaled to NRP 10th-90th percentile range of 16.8 ppb of NO_x, adjusted for ethnicity, gender, community, year of enrollment, and age; NRP exposure restricted to 11 communities. [c]Associations further adjusted for child health insurance, no food outlet within 500 m road network residence buffer, and percent poverty in the census block; all analysis restricted to 11 communities. [d]N for SHS=3164; for maternal smoking during pregnancy 3318; for NRP 2944. [e]P<0.001.

Table S4. Characteristics of participants included in analysis (with two or more BMI measurements) and not included (with one measurement).

Socio-demographic characteristics	Included (N=3318) N (%)*	Not included (N=569) N (%)*
Health insurance for child	2739 (85.0)	431 (79.2)
Foreign born child	216 (6.6)	57 (10.0)
Race/ethnicity		
African American	155 (4.7)	44 (7.3)
Asian	151 (4.5)	14 (2.5)
Hispanic White	1000 (30.1)	170 (29.9)
White, Non-Hispanic	1825 (55.0)	283 (49.7)
Other	187 (5.6)	58 (10.6)
Responding parental education		
High school or less	1055 (33.2)	211 (39.7)
Some college	1417 (44.6)	238 (44.7)
College graduate	705 (22.2)	83 (15.6)

*For the first observation of the participant (N=3318); denominator varies due to missing covariates values.

Erratum: A Longitudinal Cohort Study of Body Mass Index and Childhood Exposure to Secondhand Tobacco Smoke and Air Pollution: The Southern California Children's Health Study

McConnell R, Shen E, Gilliland FD, Jerrett M, Wolch J, Chang CC, et al. 2015. Environ Health Perspect 123(4):360–366; http://dx.doi.org/10.1289/ehp.1307031

In Table S4 of the Supplemental Material, numbers of observations (N) and corresponding percentages (%) were mislabeled as mean values (kg/m^2) ± SD. The table has been corrected, and the online PDF reflects these changes.

The authors regret the errors.
